# Intestinal colonization due to *Escherichia coli* ST131: risk factors and prevalence

**DOI:** 10.1186/s13756-018-0427-9

**Published:** 2018-11-15

**Authors:** Isabel Morales Barroso, Lorena López-Cerero, María Dolores Navarro, Belén Gutiérrez-Gutiérrez, Alvaro Pascual, Jesús Rodríguez-Baño

**Affiliations:** 1 Unidad Clínica de Enfermedades Infecciosas y Microbiología, Hospital Universitario Virgen Macarena, Seville, Spain; 2 Departamentos de Medicina y Microbiología, Universidad de Sevilla / CSIC/ Instituto de Biomedicina de Sevilla, Seville, Spain; 3 Unidad Clínica de Urgencias, Hospital Universitario Virgen Macarena, Seville, Spain

**Keywords:** *Escherichia coli*, ST131, Intestinal colonisation, Risk factors, Carriage, Prevalence colonization, Outcome

## Abstract

**Background:**

*Escherichia coli* sequence type 131 (ST131) is a successful clonal group that has dramatically spread during the last decades and is considered an important driver for the rapid increase of quinolone resistance in *E. coli*.

**Methods:**

Risk factors for rectal colonization by ST131 *Escherichia coli* (irrespective of ESBL production) were investigated in 64 household members (18 were colonized) and 54 hospital contacts (HC; 10 colonized) of 34 and 30 index patients with community and nosocomial infection due to these organisms, respectively, using multilevel analysis with a p limit of < 0.1.

**Result:**

Colonization among household members was associated with the use of proton-pump inhibitors (PPI) by the household member (OR = 3.08; 95% CI: 0.88–10.8) and higher age of index patients (OR = 1.05; 95% CI; 1.01–1.10), and among HC, with being bed-ridden (OR = 21.1; 95% CI: 3.61–160.0) and having a urinary catheter (OR = 8.4; 95% CI: 0.87–76.9).

**Conclusion:**

Use of PPI and variables associated with higher need of person-to-person contact are associated with increased risk of rectal colonization by ST131. These results should be considered for infection control purposes.

## Introduction

*Escherichia coli* is among the most frequent cause of bacterial infection in humans, particularly in the urinary and digestive tracts. Therefore, antimicrobial resistance in *E. coli* has important consequences for antibiotic use. *E. coli* sequence type 131 (ST131) is a successful clonal group that has dramatically spread during the last decades, and is considered an important driver for the rapid increase in antimicrobial resistance in *E. coli* to quinolones; also some lineages of this clone, such us *H30*Rx clade C2, have been linked to the dissemination of the extended-spectrum β-lactamases (ESBL) CTX-M-15 and CTX-M-14 [1–3]. Importantly, these isolates usually exhibit the virulence factors associated with extraintestinal pathogenic *E. coli* strains [1]. Therefore, ST131 is important because of its combination of successful spread, antibiotic resistance and virulence.

Most epidemiological studies on intestinal colonisation by ST131 has been performed on isolates producing ESBLs. However, most ST131 isolated from rectal [4] or clinical samples [5] do not produce ESBLs. Thus, investigating the epidemiology of ST131 is challenging because these isolates lack a specific susceptibility marker, so that molecular methods must be applied to a high number of *E. coli* isolates in order to identify those belonging to ST131 clonal group. This is complex for prospective studies. Therefore, our knowledge about risk factors for the acquisition of non-ESBL-producing ST131 is very limited but would be relevant from an infection control perspective. The objectives of this study were to investigate the risk factor for colonization among contacts of patients infected with ST131 *E. coli* irrespective of ESBL production, in the community and hospitals.

## Methods

The risk factors for colonisation with ST131 *E. coli* were studied using a case-control design in 34 community and 30 hospital clusters, conducted at Hospital Universitario Virgen Macarena, a tertiary hospital attending 550.000 population in Seville, Spain, from April 2012 to April 2013. The study design was previously reported [6]. Briefly, the clusters were identified from an “index patient” suffering an infection due to ST131 *E. coli* (detected by PCR for *O25b rfb*4, allele 3 of the *pabB* gene and for the B2_3_ phylogroup), and was formed by his/her contacts. Community index patients (*n* = 34) were attending the emergency department and had not been admitted to the hospital during the previous month, and nosocomial index patients (*n* = 30) were hospitalized for > 48 h when the sample was obtained. The household members of each index community index patients (*n* = 64; median per index patient, 2; range 1–4) formed the community clusters, and the patients admitted to the same or nearest rooms and attended by the same team of nurses as the nosocomial index patient (*n* = 54; median, 2; range, 1–6) formed the nosocomial clusters. Rectal colonisation by ST131 *E. coli* was studied in all participants by performing rectal swabs within one week of index case detection, and 1 and 3 months later.

The microbiological procedures were previously reported [4]; in summary, rectal swabs were inoculated to Brilliance UTI agar, MacConkey agar containing 4 mg/L cefotaxime and a blood agar plate; all distinct *E. coli* morphotypes were screened for O25b/pabB3/B23. ESBL production was studied in all third-generation cephalosporin-resistant isolates by the double-disk synergy test. Antibiotic susceptibility was studied by broth microdilution according to Clinical and Laboratory Standards Institute (CLSI) recommendations [7].

**Fig. 1:**
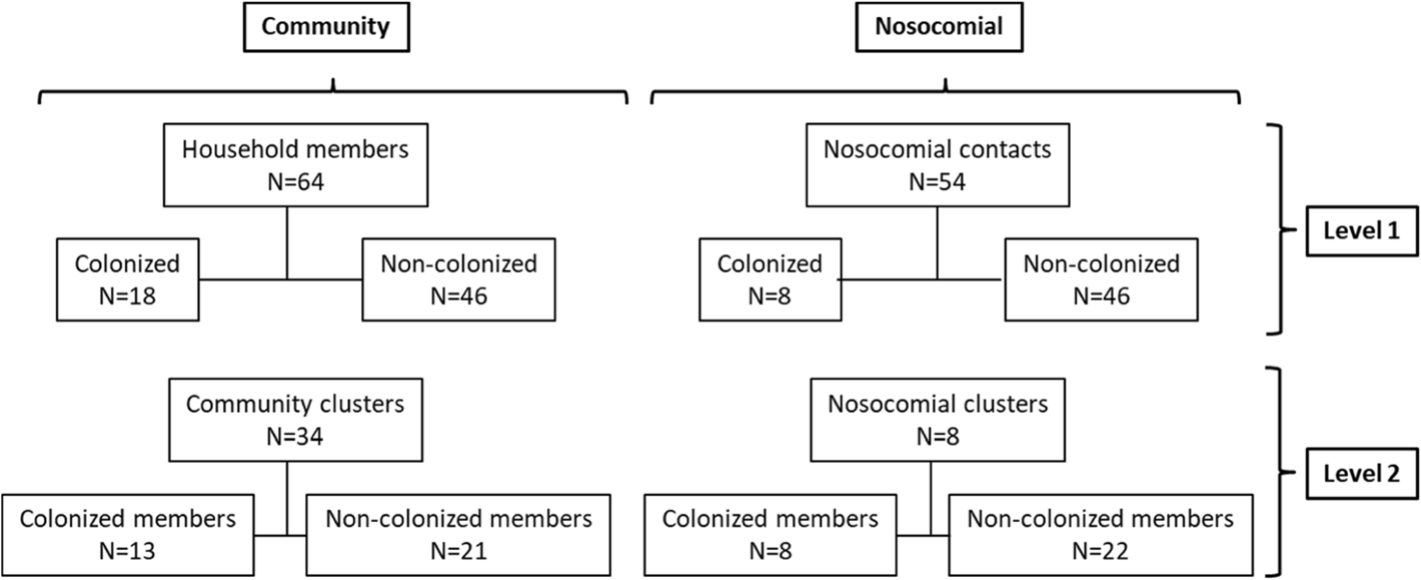
Distribution of participants

As previously reported, 18/64 (28.1%) household members from 13/34 (38.2%) community clusters and 8/54 (14.8%) contacts from 8/30 (26.6%) nosocomial clusters were colonized by ST131 *E. coli* in at least one of the 3 rectal swabs performed [4] (Fig. 1). Data were collected by personal interviews using a predesign questionnaire with the variables showed in Tables 1 and 2, before any information about colonization status was known. To consider both the individual and cluster levels of exposure to risk factors for colonisation with ST131 *E. coli*, the variance among patients in the clusters were the risk factors for colonisation with ST131 *E. coli* in any of the 3 rectal swabs were investigated using a multilevel logistic regression analysis if the variance among patients in the clusters was significant (*P* value by Wald test ≤0.2); in that case, a two-level logistic regression analysis (level 1 was formed by the individual participants and level 2 by clusters) was performed. If *p* value by Wald test was > 0.2, a conditional logistic regression was performed for level 1. Variables were kept in the models if their *p* values were < 0.1. Mlwin 3.0 (University of Bristol, UK) and SPSS 21.0 (IBM Corp, Armonk, New York, USA) were used for the analyses. The Institutional Review Board of the Hospital Universitario Virgen Macarena approved the study.

**Table 1:** Characteristics of ST131 *E. coli* colonized and non-colonized household members of index community patients. Data are expressed as number of exposed patients (percentage) except where specified

Features of contacts (individual level)	ST 131 colonized household members (*n* = 18)	Non-ST131 colonized household members (*n* = 46)	*P*	Adjusted OR (multilevel)	*p*
Median age in years (IQR)	55 (47.25–74)	48 (37–58.5)	0.06		
Male gender	6 (33.3)	17 (37.0)	0.78		
Median Charlson index (IQR)	0 (0–0)	0 (0–0)	0.42		
Diabetes mellitus	2 (11.1)	3 (6.5)	0.61		
Cancer	0	3 (6.5)	0.55		
Recurrent urinary tract infections	1 (5.6)	0	0.28		
Dependent for basic activities	2 (11.1)	0	0.07		
Bed-ridden	1 (5.6)	0	0.28		
Recurrent urinary tract infections	1 (5.6)	0	0.22		
Urinary catheter	0	0			
Usual caregiver of index patient	11 (61.1)	21 (45.6)	0.26		
Contact with farm animals	1 (5.6)	1 (2.2)	0.48		
Shared bathroom with index patient	14 (77.8)	42 (91.3)	0.14		
Travel abroad in the previous 3 months	0	1 (2.2)	1		
Sexual partner of index patient	6 (33.3)	14 (30.4)	0.82		
Mean meal outside home > 3 days/week	3 (16.7)	6 (13.0)	0.70		
Cook regularly at home	9 (50)	22 (47.8)	0.87		
Eat chicken products ≥1 per week	11 (61.1)	28 (60.9)	0.98		
Eat turkey products ≥1 per week	5 (27.8)	17 (37.0)	0.48		
Eat raw vegetables ≥1 per week	17 (94.4)	39 (84.8)	0.29		
Recent antibiotic use	3 (16.7)	1 (2.2)	0.06		
Proton pump inhibitor use	9 (50.0)	12 (26.1)	0.06	3.08 (0.88–10.8)	0.07
Features of index patient (cluster level)	Clusters with one or more ST131 colonized household member (*n* = 13)	Clusters without any ST131 colonized household member (*n* = 21)	*P*		
Median age in years (IQR)	79 (69.75–83.75)	67.5 (51.5–81)	0.16	1.05 (1.01–1.10)	0.05
Male gender	8 (61.5)	8 (38)	0.43		
Bed-ridden	0	0	0.81		
Dependent for basic activities	2 (15.3)	2 (9.5)	0.65		
Urinary catheter	4 (30.7)	3 (14.28)	0.36		
Median Charlson index (IQR)	0.5 (0–2)	1.5 (0–2)	1		
Pets at home	7 (53.8)	11 (52.4)	0.96		
Recent antimicrobial use	9 (69.2)	13 (61.9)	0.84

**Table 2:** Characteristics of ST131 *E. coli* colonized and non-colonized hospital contacts of index nosocomial patients. Data are expressed as number of exposed patients (percentage) except where specified

Features of contacts (individual level)	ST 131 colonized hospital contacts (*n* = 10)	Non-ST131 colonized hospital contacts (*n* = 44)	*P*	Adjusted OR	*p*
Median age in years (IQR)	81.5 (75.5–87.75)	6.08 (59.75–80.0)	0.05		
Male gender	5 (50.0)	20 (45.4)	0.79		
Median Charlson index (IQR)	1 (0.25–5)	1 (0–3.75)	0.76		
Diabetes mellitus	3 (30.0)	15 (34.0)	0.80		
Cancer	3 (30.0)	13 (29.5)	0.98		
Liver cirrosis	0	1 (2.2)	1		
Recurrent urinary tract infections	1 (10.0)	1 (2.2)	0.34		
Dependent for basic activities	5 (50.0)	3 (6.8)	0.003	21.1 (3.61–160.0)	0.001
Bed-ridden	1 (10.0)	0	0.18		
Shared room with index patient	7 (70.0)	24 (54.5)	0.37		
Surgery during present admission	4 (40.0)	15 (34.0)	0.72		
Urinary catheter	6 (60.0)	12 (27.2)	0.19	8.4 (0.97–76.9)	0.05
Recent antimicrobial use	4 (40.0)	16 (36.3)	0.83		
Proton pump inhibitor use	6 (60.0)	34 (77.2)	0.85		
Median (IQR) days of hospital stay	5.5 (4.0–11.25)	6 (3.75–9)	0.70		
Features of index patient (cluster level)	Clusters with at least one ST131 colonized hospital contact (*n* = 8)	Clusters without any ST131 colonized hospital contact (*n* = 22)	*P*		
Median age in years (IQR)	62.50 (52.75–78)	67.10 (58.25–80.75)	0.70		
Male gender	5 (62.5)	6 (27.3)	0.28		
Median Charlson index (IQR)	2.60 (0–6)	2.40 (0–6)	0.7		
Bed-ridden	0	3 (13.6)	0.53		
Dependent for basic activities	2 (25)	4 (18.1)	0.73		
Admission to a surgical ward	4 (50)	8 (36.3)	0.66		
Admission to a medical ward	5 (62.5)	11 (50)	0.74		
Admission to an intensive care unit	1 (12.5)	0	0.21		
Surgery during present admission	4 (50)	5 (22.7)	0.39		
Urinary catheter	7 (87.5)	10 (45.4)	0.76		
Recent antimicrobial use	7 (87.5)	10 (45.4)	0.42		
Median (IQR) days of hospital stay	27.2 (7–46)	35.5 (14.75–24)	0.59		

## Results

The distribution of participants according to colonization status and clusters is shown in Fig. 1. The univariate comparison in exposure to potential risk factors between the 18 colonized and 46 non-colonized participants from community clusters, and between community clusters with and without colonized member (13 and 21, respectively) are shown in Table 1. The variables with a *p* value < 0.2 for their association to colonization were higher age, being dependant for basic activities, not sharing bathroom with index case, recent antibiotic use and proton pump inhibitors (PPI) use in the individual level, and age of the index patient in the cluster level. The p value for the variance at the cluster level was 0.20; therefore, a multilevel analysis was performed; the variables associated with ST131 colonisation were PPI use in the individual level and age of the index patient in the cluster level (Table 1).

The univariate comparison in exposure to potential risk factors between the 10 colonized and 44 non-colonized participants from nosocomial clusters, and between nosocomial clusters with and without a colonized member (8 and 22, respectively) are shown in Table 2. The variables with a *p* value < 0.2 for their association to colonization in the individual level were age, being dependant for basic activities or bed-ridden and having a urinary catheter; no variable showed was selected in the cluster level. The p value for the variance at the cluster level was 0.95; therefore, multilevel analysis was not performed; the variables associated with colonisation in the multivariate conditional logistic regression were being dependent for activities and urinary catheter (Table 2).

The susceptibility of ST131 isolates is shown in Table 3. Overall, 6 isolates (21.4%) were ESBL-producers.

**Table 3:** Antimicrobial susceptibility of ST131 *Escherichia coli* isolates. Data are number of susceptible isolates (percentage)

Antimicrobial	All isolates (*n* = 28)	Isolates from household members (n = 18)	Isolates from hospital contacts (n = 10)
Ampicillin	6 (21.4)	6 (33.3)	0
Amoxicillin-clavulanic acid	9 (31.2)	9 (50)	0
Piperacillin-tazobactam	27 (96.4)	18 (100)	9 (90)
Ceftriaxone	22 (78.5)	15 (83.3)	7 (70)
Ceftazidime	22 (78.5)	15 (83.3)	7 (70)
Ertapenem	28 (100)	18 (100)	10 (100)
Ciprofloxacin	9 (32.1)	6 (33.3)	3 (30)
Gentamicin	24 (85.7)	17 (94.4)	7 (70)
Tobramycin	19 (67.8)	14 (77.8)	5 (50)
Amikacin	28 (100)	18 (100)	10 (100)
Fosfomycin	28 (100)	18 (100)	10 (100)

## Discussion

Despite the obvious limitation related to low numbers, we were able to identify some risk factors for rectal colonisation by ST131 in community and nosocomial clusters of patients with infection due to these organisms. PPI use and higher age in the index patients are associated with intestinal colonisation with ST131 among household members of patients with previous community-acquired infection due to this organism; this is interesting as PPI use has been found also to be a risk factor for colonisation with ESBL-producers [8] and other enteric pathogens. This might be related to the fact that PPI eliminate the barrier that the acid content of the stomach pose to digestive tract colonisation of exogenous bacteria by ingestion. The higher age in index patients may be interpreted as higher need for care and therefore more frequent contact. In hospitals, the risk factors found (dependence for basic activities and urinary catheter) might also be associated with increased need for care and contact. These results suggest that direct contact would be a prominent mechanism of transmission for ST131 and build on the concept that avoiding such direct person-to-person transmission would be critical to reduce the spread of these isolates [9].

The information about risk factors for colonisation with ST131 is scant. While household transmission of ST131 isolates has been well demonstrated [6, 10], we could find no studies investigating the risk factors for transmission. In healthcare centers, Han et al. could not identify relevant differences between 29 and 8 long-term care facility (LTCF) residents colonized with ST131 and non-ST131 fluoroquinolone-resistant *E. coli*. [11]; in another study in a LTCF, Burgess et al. found that time of admission, being unable to sign consent, decubitus ulcer and fecal incontinence were risk factors for colonisation with ciprofloxacin-resistant ST131 *E. coli* [12]. Other studies only investigated ESBL-producing ST131 isolates [1, 2].

Previous antibiotic use was not identified as risk factor in those studies or in the present one; however, we found exposure to antibiotics (specifically amoxicillin-clavulanic acid and fluoroquinolones) to be associated with increased risk of infection due to ST131 [13]. While it may just be a problem of statistical power, antibiotics might be more important in the case of infections by selecting ST131 in the gut of already colonized persons than as a factor clearly favouring colonization.

This study has limitations that should be considered, including a limited statistical power to detect risk factors due to small sample size in relation with the difficulties in performing a study on colonization with bacteria for which no phenotypic marker exists; the sensitivity of detection of ST131 from rectal swabs might be lower than desired; and the results might be applicable only to clusters with an infected person and areas with a similar epidemiology and clades of ST131.

## Conclusion

In conclusion, use of PPI and variables associated with higher need of person-to-person contact are associated with increased risk of rectal colonization by ST131. These results should be considered for infection control purposes.

## Abbreviations

ESBL: extended-spectrum beta-lactamase
LTCF: long-term care facility
PPI: proton-pump inhibitors
ST131: *Escherichia coli* sequence type 131
